# Effects of PTH glandular and external dosing patterns on bone cell activity using a two-state receptor model—Implications for bone disease progression and treatment

**DOI:** 10.1371/journal.pone.0283544

**Published:** 2023-03-30

**Authors:** Denisa Martonová, Maxence Lavaill, Mark R. Forwood, Alexander Robling, David M. L. Cooper, Sigrid Leyendecker, Peter Pivonka

**Affiliations:** 1 Mechanical, Medical and Process Engineering, Queensland University of Technology, Brisbane, Queensland, Australia; 2 Institute of Applied Dynamics, Friedrich-Alexander-Universität Erlangen-Nürnberg, Erlangen, Germany; 3 School of Pharmacy and Medical Sciences, Griffith University, Gold Coast, Queensland, Australia; 4 Anatomy, Cell Biology & Physiology, School of Medicine, Indiana University, Indianapolis, Indiana, United States of America; 5 Department of Anatomy, Physiology and Pharmacology, University of Saskatchewan, Saskatoon, Canada; Central State University, UNITED STATES

## Abstract

Temporal aspects of ligand specificity have been shown to play a significant role in the case of pulsatile hormone secretion, as exemplified by parathyroid hormone (PTH) binding to its receptor (PTH1R), a G-protein-coupled receptor expressed on surfaces of osteoblasts and osteocytes. The latter binding reaction regulates intracellular signalling and subsequently modulates skeletal homeostasis via bone remodelling. PTH glandular secretion patterns dictate bone cellular activity. In healthy humans, 70% of PTH is secreted in a tonic fashion, whereas 30% is secreted in low-amplitude and high-frequency bursts occurring every 10–20 min, superimposed on the tonic secretion. Changes in the PTH secretion patterns have been associated with various bone diseases. In this paper, we analyse PTH glandular secretion patterns for healthy and pathological states and their link to bone cellular responsiveness (*α*_*R*_). We utilise a two-state receptor ligand binding model of PTH to PTH1R together with a cellular activity function which is able to distinguish various aspects of the stimulation signal including peak dose, time of ligand exposure, and exposure period. Formulating and solving several constrained optimisation problems, we investigate the potential of pharmacological manipulation of the diseased glandular secretion and via clinical approved external PTH injections to restore healthy bone cellular responsiveness. Based on the mean experimentally reported data, our simulation results indicate cellular responsiveness in healthy subjects is sensitive to the tonic baseline stimulus and it is 28% of the computed maximum responsiveness. Simulation results for pathological cases of glucocorticoid-induced osteoporosis, hyperparathyroidism, initial and steady state hypocalcemia clamp tests indicate *α*_*R*_ values significantly larger than the healthy baseline (1.7, 2.2, 4.9 and 1.9-times, respectively). Manipulation of the pulsatile glandular secretion pattern, while keeping the mean PTH concentration constant, allowed restoration of healthy baseline values from these catabolic bone diseases. Conversely, PTH glandular diseases that led to maximum bone cellular responsiveness below the healthy baseline value can’t be restored to baseline via glandular manipulation. However, external PTH injections allowed restoration of these latter cases.

## 1 Introduction

Calcium is the most abundant mineral in all vertebrates [1]. Most of the body’s calcium (99%) is stored in the bones and teeth, with only 1% stored in blood and other tissues. Calcium serves as a critical regulator of multiple physiological processes, including blood coagulation, nerve conduction, membrane permeability, muscle contraction, enzyme activity and hormone release [2]. Therefore, maintenance of extracellular fluid (ECF) free calcium concentrations in the normal physiological range is tightly regulated in all organisms [3].

As outlined in the recent review by Hernandez-Castellano et al. [1], the classic calciotropic hormones—parathyroid hormone (PTH), calcitriol and calcitonin—regulate calcium homeostasis under normal physiological conditions in mammals by coordinating the availability of ECF free calcium in the blood at the level of the bones, intestine and kidneys. In humans ECF free calcium is regulated by dynamic secretion of PTH by the parathyroid gland and to a lesser contribution from calcitriol and calcitonin [4].

In the following, we focus on PTH secretion patterns of the human parathyroid gland which targets the parathyroid hormone/parathyroid hormone-related protein receptor (PTH/PTHrP type 1 receptor), also commonly known as PTH1R. As highlighted in the review of Cheloha et al. [5], PTH1R is a G-protein-coupled receptor (GPCR) that regulates skeletal development, bone turnover and mineral homeostasis. PTH1R transduces stimuli from PTH and PTH-related-peptide (PTHrP) into the interior of target cells (i.e. cells of the osteoblastic lineage) to promote several divergent signalling cascades. This receptor is able to exist in at least 2 distinct conformation states (R0 and RG) that differ in their signalling response [6]. Ligands that bind selectively to the RG state result in a shorter signalling response, whereas ligands that bind selectively to the R0 state result in prolonged signalling response [7]. The downstream cyclic adenosine monophosphate (cAMP) signalling response is considered to be the primary signalling cascade that mediates the effect of the PTH receptor. PTH1R can modulate a ligand’s biological activity depending of the preferential binding to either state [7]. Since most PTH analogues have some affinity for both states of the PTH/PTHrP receptor, the behaviour of a given PTH vis à vis duration of effect is determined by the ratio of binding affinities to each of these receptor states.

As reviewed by Chiavistelli et al. [8], PTH secretion is characterised by an ultradian rhythm with tonic and pulsatile components. In healthy subjects, the majority of PTH is secreted in a tonic fashion (70%), whereas approximately 30% is secreted in low-amplitude and high-frequency bursts occurring every 10–20 min, superimposed on the tonic secretion. Changes in the ultradian PTH secretion pattern have been associated with various diseases including primary and secondary osteoporosis, and hyperparathyroidism. Depending on the dual modifications of pulsatile and tonic components the latter diseases may have different severity of skeletal complications. Furthermore, the pulsatile component can be selectively activated as part of the PTH glandular immediate response to changes in ECF free calcium concentrations [9]. Acute hypocalcemia induces a selective, several-fold increase in bursts frequency and amplitude, whereas hypercalcemia suppresses the PTH pulsatile secretion component, as does prolonged calcitriol therapy.

In the mid 80’s, the development of mathematical tools such as heuristic pulse detection methods including Pulsar [10], Cluster [11] and the algorithm of Santen and Bardin [12], allowing decomposition of hormonal release patterns into tonic and pulsatile components, has paved the way for identifying the biological importance of dynamic hormonal secretion. Current theory suggests that biological information in hormonal systems is encoded as a dynamic hormone concentration in the circulating blood. Variations of the concentration are determined by glandular secretion rate and hormonal metabolism. It is now well established that most hormones exhibit a dynamic pattern of episodic secretory and metabolic events. Furthermore, cell biology experiments suggest that pulsatile secretory patterns govern most appropriately the dynamic modulation of the hormone-receptor-interaction, namely receptor de- and resensitisation.

Endocrine diseases are typically defined by comparing serum levels of endocrine factors with the ‘normal (or reference) range’. This reference range is used to discern hyper- and hypofunction of respective glands. Dynamic diseases evolve within the normal range and are characterised by increased or decreased secretory dynamics [8]. These high or low dynamic functional states govern cellular responses at the target organs and a disturbed function of a gland can lead to development of progressive diseases. Development of skeletal diseases such as osteoporosis might be linked to the PTH1R signalling pathway and, consequently, it is crucial to understand the dynamics of PTH-PTH1R binding and subsequent signal transduction. Furthermore, osteoporosis therapies such as intermittent PTH(1–34) can also be targeted to PTH1R signalling inducing an osteoanabolic effect [13].

In this paper we utilise a two-state receptor model of PTH-PTH1R originally proposed in [14]. This model is an extension of the work of Potter et al. [15] with respect of introducing a (osteoblastic) *cellular responsiveness* function which is able to distinguish different dynamic PTH dosing patterns. The *cellular responsiveness* has been originally proposed by Li and Goldbeter for pulsatile (square-wave) stimuli [16] applied to gonadotropin-releasing hormone. Using the experimental data of Harms et al. [17, 18] of PTH glandular secretion patterns, we first investigate the reference range of cellular activity representing normal bone homeostasis. We then introduce perturbations of both the tonic and pulsatile components of PTH secretion to mimic various disease states including osteoporosis and hyperparathyroidism. To assess the possibility to achieve a certain *cellular responsiveness* for a given dynamic PTH dosing pattern, we solve a constrained optimisation problem, where the area under the curve (AUC) representing the evolution of the PTH concentration in time (i.e. the mean PTH concentration) is kept constant. These simulations are motivated by the fact that drugs that target calcium-sensing receptors on parathyroid cells can directly affect PTH glandular secretion patterns [19, 20].

In a subsequent analysis, we investigate the effects of an external PTH dose in the form of a subcutaneous (sc) injection on the *cellular responsiveness* for a given disease state. The latter simulations are motivated by the fact that intermittent PTH sc daily injections are used for osteoporosis treatment. The additional PTH dose contributes to an additional *cellular responsiveness* and so has the ability to modify bone cellular activity. We first use the currently clinically approved dose of 20*μ*g daily PTH injections and subsequently, we optimise the dosing pattern to obtain firstly, the maximal *cellular responsiveness* and secondly, the *cellular responsiveness* of a healthy person.

In contrast to previous works [14, 16] presenting an analytical solution for a special case of the optimisation of the *cellular responsiveness*, we make use of numerical approximation methods. The numerical approach is much more flexible in the sense that there are no special case restrictions on the parameters in the model and one can easily consider different scenarios by formulation constrained optimisation problems with various objective functions and constraints.

In Section 2, we introduce the two-state receptor ligand binding model for PTH. This section includes an introduction of various theoretical measures of bone cellular activity (Subsection 2.1) and a representation of PTH plasma concentration (Subsection 2.2). In Section 3, the considered optimisation problems are introduced. In Sections 4 and 5, the results are presented and discussed.

## 2 Two-state receptor ligand binding model for PTH

Receptor kinetics models have been developed for many different receptor systems, ranging from general models for entire classes of receptors to specific models for a particular receptor expressed in a specific type of cell (see [21, 22] for reviews of existing models). GPCRs such as the PTH1R, are known to exist in various conformations that have different affinities for processes such as binding, activation, and phosphorylation [23, 24]. It has been shown that PTH1R undergoes multiple conformational changes as it binds to ligands and becomes activated [25, 26]. Most commonly, these multiple conformational states can be conceptually grouped into two functional states: active and inactive. A widely accepted model for the activation of GPCRs is the two-state receptor ligand binding model first proposed by Segel et al. in the study of exact sensory adaptation [14]. This model was subsequently employed by Li and Goldbeter to study frequency specificity in intercellular communication [16] and further discussed in [27].

**Fig 1:**
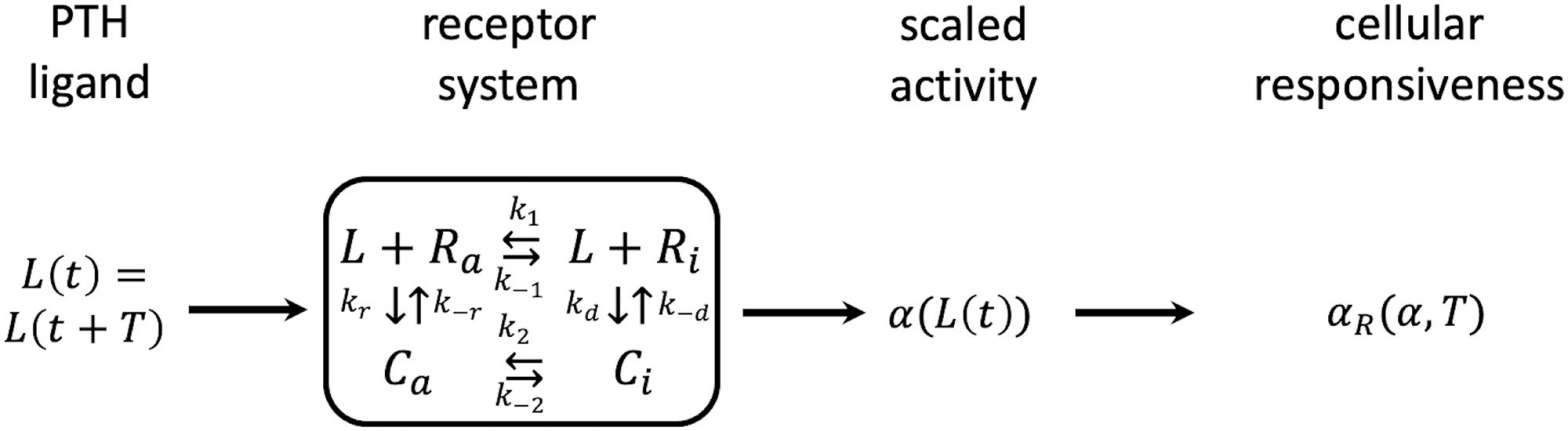
Schematic of the two-state receptor model representing PTH to PTH1R binding based on [16]. *L*(*t*) = PTH concentration; *t* = time; *T* = period; *R*_*a*_ = active receptor concentration; *R*_*i*_ = inactive receptor concentration; *C*_*a*_ = active ligand-receptor complex concentration, *C*_*i*_ = inactive ligand-receptor complex concentration, *k*_±*i*_ = kinematic parameters (*i* ∈ {1, 2, *d*, *r*}); *α* = scaled activity; *α*_*R*_ = *cellular responsivenes*.

Based on the ability of PTH1R to change conformation independent of a ligand, it is assumed that the two receptor conformational states can transform into each other regardless of its binding to its ligand. This can be achieved either through covalent modification of PTH1R or through simple conformational change. In either case, these correspond to active (or nondesensitised) receptor states, *R*_*a*_, and inactive (or desensitised) receptor states, *R*_*i*_, which differ by their capability of eliciting a cellular response upon binding of the ligand. Both receptor states combine with the PTH ligand *L* to form active and inactive ligand-receptor complexes *C*_*a*_ and *C*_*i*_ (see Fig 1 for a schematic of the model kinetics). This creates a distribution among the four receptor species with the total concentration of receptor *R*_*T*_(= [*R*_*a*_] + [*C*_*a*_] + [*C*_*i*_] + [*R*_*i*_]).

The ordinary differential equations (ODEs) for the two-state receptor model can be summarised in matrix notation as [14]:

$$\begin{array}{c} \frac{d\text{C}}{dt}=\text{K}\left(L\left(t\right)\right)\text{C}(t), \end{array}$$ (1)

where the vector of unknown time-dependent concentration C(t) for the receptor states is given in its scaled form as

$$\begin{array}{c} \text{C}=[\begin{array}{c} r_{a}\\ c_{a}\\ c_{i}\\ r_{i} \end{array}], \end{array}$$ (2)

with *r*_*a*_ = *R*_*a*_/*R*_*T*_, *c*_*a*_ = *C*_*a*_/*R*_*T*_, *c*_*i*_ = *C*_*i*_/*R*_*T*_, and *r*_*i*_ = *R*_*i*_/*R*_*T*_. The constant coefficient matrix describing the ligand-receptor binding kinetics is given as

$$\begin{array}{c} \text{K}\left(L\right)=[\begin{array}{cccc} -k_{1}-k_{r}L & k_{-r} & 0 & k_{-1}\\ k_{r}L & -k_{2}-k_{-r} & k_{-2} & 0\\ 0 & k_{2} & -k_{-2}-k_{-d} & k_{d}L\\ k_{1} & 0 & k_{-d} & -k_{-1}-k_{d}L \end{array}] \end{array}$$ (3)

see Table 1 for details on the specific parameters. As the above coefficient matrix has not full rank, the system of ODEs reduces to

$$\begin{array}{c} \left[\begin{array}{c} {\dot{r}}_{a}\\ {\dot{c}}_{a}\\ {\dot{c}}_{i} \end{array}]=[\begin{array}{cccc} -k_{1}-k_{r}L & k_{-r} & 0 & k_{-1}\\ k_{r}L & -k_{2}-k_{-r} & k_{-2} & 0\\ 0 & k_{2} & -k_{-2}-k_{-d} & k_{d}L \end{array}][\begin{array}{c} r_{a}\\ c_{a}\\ c_{i}\\ 1-(r_{a}+c_{a}+c_{i}) \end{array}\right] \end{array}$$ (4)

with the conservation condition

$$\begin{array}{c} r_{a}+c_{a}+c_{i}+r_{i}=1 \end{array}$$ (5)

being automatically fullfilled by solution of Eq 1. In Eqs (3) and (4), *L* denotes the ligand concentration, i.e. PTH in plasma (or bone fluid) which binds to PTH1R expressed on osteoblastic cells. We note that, unlike in the paper of [15], we do not compute the PTH concentration from an additional ODE. We assume that this can be approximated by a simple square-wave stimulus characterised by the the peak ligand concentration *γ*_1_, duration of the on-phase *τ*_1_ and period *T*, see [16]. For the physiological gland pulses, these quantities are derived from experimental data, whereas for the PTH injection, we compute the PTH concentration in plasma from a one compartment pharmacokinetic (PK) model of PTH sc injection in humans [28]. This model delivers the periodic ligand stimulus, *L*(*t*) = *L*(*t* + *T*). Binding of the ligand to the respective receptor induces a cellular response. As was pointed out by Segel and co-workers [14], the nature of this response as well as the precise manner by which it is linked to ligand binding differ from one particular system to another. In the context of PTH binding to its receptor, PTH1R, the dissociation constant is approximately 1nM [15, 29, 30]. For the kinetic parameters *k*_1_, *k*_−1_, *k*_2_, *k*_−2_, we adopted the experimentally derived parameters published by [31] for cAMP signalling which uses a GPCR, too. Table 1 summarises the model parameters used for the two-state receptor model.

**Table 1:** Parameter values for the two-state receptor model of PTH-PTH1R as illustrated in Fig 1.

Parameter	Rate constant description	Value	Source
*k*_1_	conversion receptors: *R*_*a*_ → *R*_*i*_	0.012 min^−1^	[31]
*k*_−1_	conversion receptors: *R*_*i*_ → *R*_*a*_	0.104 min^−1^	[31]
*k*_2_	conversion complexes: *C*_*a*_ → *C*_*i*_	0.222 min^−1^	[31]
*k*_−2_	conversion complexes: *C*_*i*_ → *C*_*a*_	0.055 min^−1^	[31]
*K*_*r*_ = *k*_−*r*_/*k*_*r*_	dissociation constant for active complex	1 nM	[29, 30]
*K*_*d*_ = *k*_−*d*_/*k*_*d*_	dissociation constant for inactive complex	10^3^ nM	condition of detailed balance [16]

### 2.1 Definition of bone cellular responses: Activity functions

Bone cellular response is induced by binding of PTH to PTH1R. Following the work of Segel et al. [14], we assume that the effect induced by the PTH stimulus is measured by a quantity, the so-called scaled activity *α*, which can be defined as a weighted linear combination of receptors and complexes normalised concentrations as [14]:

$$\begin{array}{c} \alpha \left(L\right)=a_{1}r_{a}+a_{2}c_{a}+a_{3}c_{i}+a_{4}r_{i}, \end{array}$$ (6)

where *a*_*i*_ can be viewed as association constants and are hence non-negative [14]:

$$\begin{array}{c} a_{i}\geq 0,\;i=1,2,3,4 \end{array}$$ (7)

The activity defined in Eq (6) should be regarded as a loose measure of how strongly ligand binding to the receptor is contributing to the induction of a physiological cell response.

The choice of affinity coefficients (*a*_*i*_) is not arbitrary, but based on a particular biological system response. Here, we utilise the *exact adaptation* response defined by Segel et al. [14] as the systems response to a constant stimulus. For the case of *exact adaptation*, the scaled activity *α* returns to the same value at steady state regardless of the concentration of constant stimulus. The coefficients *a*_1_, *a*_3_ and *a*_4_ are chosen according to [16] with the scaling factor *s* = 100 due to smaller concentration of PTH compared to that work. *a*_2_ is computed such that the exact adaptation condition is satisfied, i.e. *a*_2_ = ((*a*_1_*K*_1_ + *a*_4_)/(*K*_1_ + 1)(*K*_2_ + 1) − *a*_3_)/*K*_2_ with *K*_1_ = *k*_−1_/*k*_1_ and *K*_2_ = *k*_−2_/*k*_2_ [14].

#### 2.1.1 Dose-response curves for periodic stimuli

As discussed in detail in [16], the aim is to define a meaningful measure of activity for the long-term effect of periodic stimuli. Implicitly, we assume that the cell response can be linked to this quantity. Here, we consider the case of *exact adaptation* for which the steady state value of activity (*α*_*s*_) equals the basal activity value (*α*_0_). Under these conditions, the basal activity *α*_0_ provides a unique reference value.

#### 2.2.2 Cellular responsiveness

As pointed out in [16], the definition of *cellular responsiveness* is incomplete in terms of the integrated activity $\alpha_{T}≔\int_{0}^{T}(\alpha \left(t\right)-\alpha_{0})\text{d}t$ from a physiological point of view. *Cellular responsiveness* should take into account the magnitude of integrated activity as well as the number of activity pulses in a given time interval. Li et al. [16] define the *cellular responsiveness* as the product of two terms:

$$\begin{array}{c} \alpha_{R}=\frac{\alpha_{T}}{\alpha_{T_{step}}}\frac{\alpha_{T}}{T} \end{array}$$ (8)

where $\alpha_{T_{step}}$ is defined as the integrated activity in response to a corresponding step increase in ligand from *γ*_0_ to *γ*_1_:

$$\begin{array}{c} \alpha_{T_{step}}\left(\gamma_{0},\gamma_{1}\right)=\tau_{a}\left(\gamma_{1}\right)\alpha_{M_{step}}\left(\gamma_{0},\gamma_{1}\right) \end{array}$$ (9)

In Eq (9), $\alpha_{M_{step}}$ denotes the maximum activity in response to a step increase in ligand from *γ*_0_ to *γ*_1_, while *τ*_*a*_(*γ*_1_) denotes the adaptation time to a constant stimulus *γ*_1_. The first term in Eq (8) is related to the magnitude of the integrated activity, i.e. $\alpha_{T}/\alpha_{T_{step}}$, which scales the integrated activity during one pulse of the periodic stimulus with respect to the integrated activity corresponding to a step increase in ligand of the same amplitude. Note $\alpha_{T_{step}}\gg \alpha_{T}$. The second term is the period (or intrinsic) average of the integrated activity *α*_*T*_/*T*, which takes not only the on-phase *τ*_1_, but also the off-phase *τ*_0_ into account. As noted in [16], the latter quantity will equate to a total integrated activity *nα*_*T*_ over a certain time interval *t* = *nT*.

### 2.2 PTH ligand concentration in plasma

We note the subscript and superscript *gl* refers to quantities connected to PTH gland secretion.

#### 2.2.1 Physiological parathyroid gland secretion

**Fig 2:**
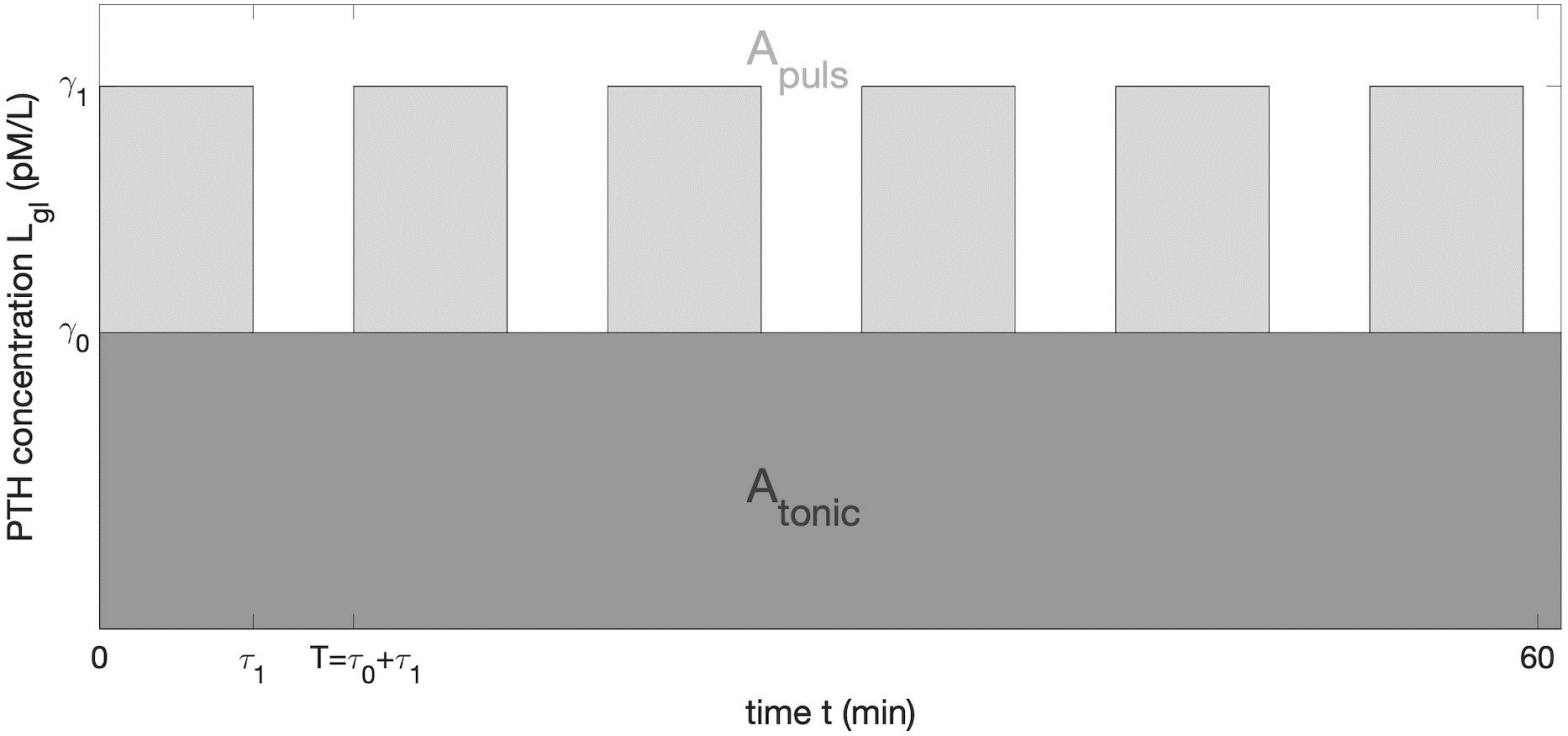
Schematic representation of the square-wave PTH ligand concentration *L*_*gl*_ (in plasma) vs. time. *γ*_1_ is the peak PTH concentration due to pulsatile glandular secretion, *γ*_0_ is the PTH concentration due to tonic glandular secrection, and *τ*_0_ and *τ*_1_ are the off- and on-phases of pulsatile component. Dark grey area *A*_*tonic*_ represents the plasma concentration resulting from a tonic secretion whereas the light grey area *A*_*puls*_ represents the plasma concentration resulting from the pulsatile secretion.

In humans, plasma PTH fluctuates periodically at a frequency of 3–7 bursts per hour, see S1 Table. Approximately 30% of circulating PTH results from pulsatile secretion and the remaining 70% from tonic secretion [32–34]. The plasma PTH concentration in the latter studies is approximated by a convolution model [35]. In the present study, however, we follow the simplified approach from [14, 16] in which the plasma ligand concentration *L*_*gl*_ is approximated by a square-wave stimulus as

$$\begin{array}{c} L_{gl}\left(\tau_{1},T,\gamma_{0},\gamma_{1},t\right)=\{\begin{array}{c} \gamma_{1}\;\text{if}\;\left(n-1\right)T\leq t\leq \left(n-1\right)T+\tau_{1}\\ \gamma_{0}\;\text{if}\;\left(n-1\right)T+\tau_{1}\leq t\leq \left(n-1\right)T+\tau_{1}+\tau_{0} \end{array} \end{array}$$ (10)

where *n* = 1, 2, 3, …, *N* represent successive pulses in the repetitive square-wave stimulation. *τ*_0_ and *τ*_1_ denote the off- and on-phase of the signal respectively, while the period *T* is defined as *T* = *τ*_0_ + *τ*_1_, see Fig 2. We note that as the equation system Eq (1) is solved numerically, in contrast to the original work [14], there is no need to introduce dimensionless signal function. Hence, *γ*_1_ and *γ*_0_ denote the concentrations (in pM/L) of PTH in plasma. Further, the model for the ligand concentration is not restricted to a square-wave stimulus and more complex functions can be used. In order to conserve the dose input signal [36], commonly referred to as AUC, we introduce the integrated concentration per an arbitrary time interval [0, *s*] as

$$\begin{array}{c} A=A_{tonic}+A_{puls}=\int_{0}^{s}L_{gl}\left(\tau_{1},T,\gamma_{0},\gamma_{1},t\right)\;\text{d}t, \end{array}$$ (11)

where *A*_*tonic*_ and *A*_*puls*_ are the AUC of the tonic and pulsatile secretions of PTH, respectively and their ratio is given as $r=\frac{A_{puls}}{A}$.

The mean plasma concentration is assumed to be ${\bar{L}}_{gl}=1/T\int_{0}^{T}L_{gl}\left(t\right)\text{d}t$. As the quantity of our interest is the plasma PTH concentration, see Eq (1), in the subsequent numerical examples, the experimental data from [17, 18] are used, see S1 Table. In these studies, the total plasma concentration was measured.

#### 2.2.2 Additional drug administration

**Fig 3:**
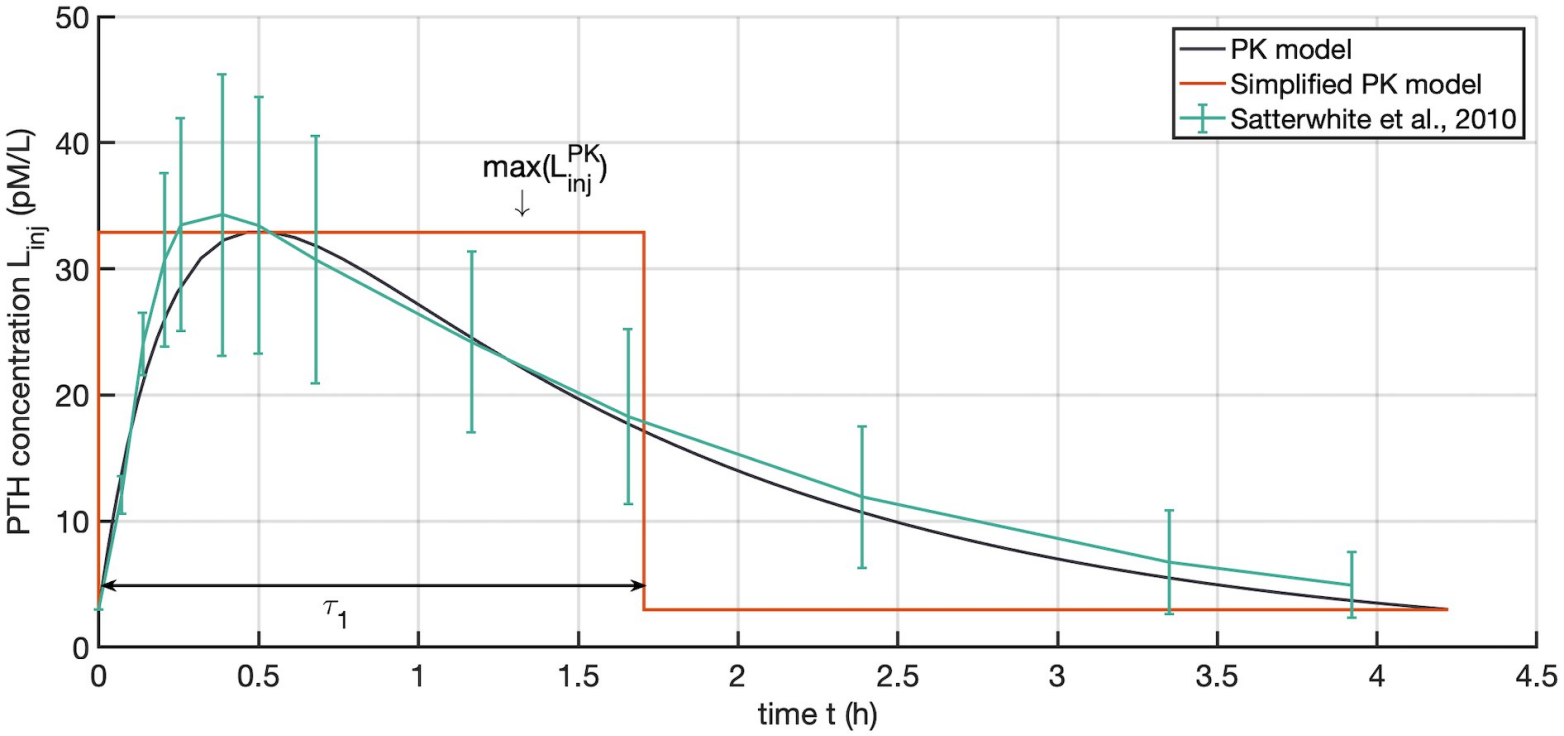
PTH plasma concentration obtained from the PTH PK model and the simplified square-wave stimulus characterised by the maximum PTH concentration $\text{max}(L_{inj}^{PK})$ and the on-phase *τ*_1_.

We note the subscript and superscript *inj* refers to quantities connected to an external sc PTH injection. An additional time-dependent PTH dose *D*(*t*) can be added in form of a sc injection. To model the resulting plasma concentration, we follow the approach taken by Pivonka and co-workers, i.e. we use a one-compartment PK model of PTH [28, 37] which is given by two ODEs

$$\begin{array}{c} \frac{\text{d}D}{\text{d}t}=-k_{a}FD\left(t\right)\;\;\text{and}\;\;\frac{\text{d}L_{inj}^{PK}}{\text{d}t}=FV_{d}k_{a}D\left(t\right)-k_{e}L_{inj}^{PK}\left(t\right), \end{array}$$ (12)

where $L_{inj}^{PK}$ is the ligand plasma concentration, the absorption rate constant *k*_*a*_ represents the drug absorption process from the sc site of injection into the blood stream, and the elimination rate constant *k*_*e*_ represents the drug elimination process from blood, *V*_*d*_ is the distribution volume and *F* is the bioavailability. Fig 3 shows PTH plasma concentration for the non-linear case and the simplified square-wave stimulus.

Assuming that the peak PTH drug concentration is the same for the square-wave stimulus and the PK model and assuming that the AUC in the (*L*, *t*)-plots representing the integrated drug concentration are equal between these two models ($A_{inj}^{PK}=A_{inj}$), we can calculate the on-phase of the square-wave signal as

$$\begin{array}{c} \tau_{1}=\frac{A_{inj}^{PK}}{\text{max}\left(L_{inj}^{PK}\right)-\gamma_{0}}, \end{array}$$ (13)

where *γ*_0_ is the PTH concentration resulting from the tonic secretion, see Eq (10). The PTH drug concentration *L*_*inj*_ can be then approximated as

$$\begin{array}{c} L_{inj}\left(D,T,\gamma_{0},t\right)=\{\begin{array}{cc} \gamma_{1}\; & \text{if}\;\left(n-1\right)T\leq t\leq \left(n-1\right)T+\tau_{1}\\ \gamma_{0}\; & \text{if}\;\left(n-1\right)T+\tau_{1}\leq t\leq \left(n-1\right)T+\tau_{1}+\tau_{0} \end{array} \end{array}$$ (14)

**Fig 4:**
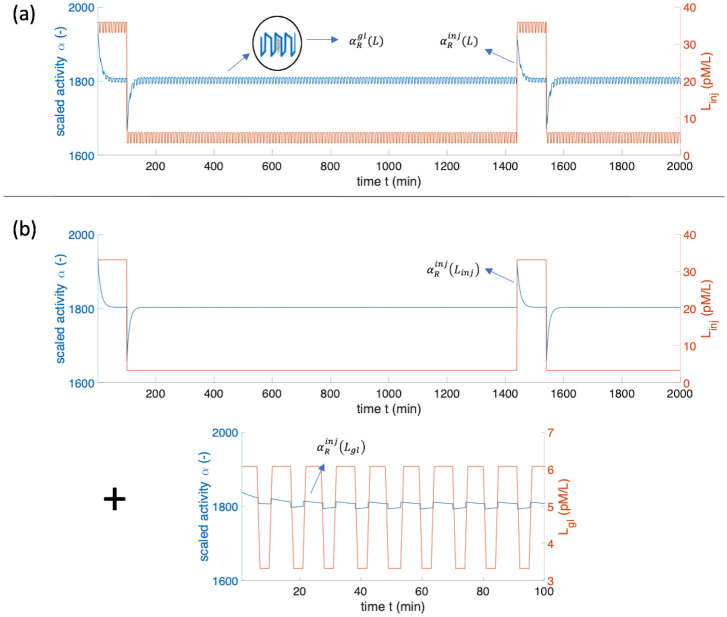
Computational simulation results of two models used to compute scaled activity *α* and *cellular responsiveness α*_*R*_ for the case of combined PTH glandular secretion and external sc daily PTH injection (*D* = 20*μ*g). (a) refers to model 1 whereas (b) refers to model 2.

To compute the resulting *cellular responsiveness*, two models are investigated:

- model 1: we assume that the resulting PTH ligand concentration in Eq (1) is *L* = *L*_*gl*_ + *L*_*inj*_, solve Eq (1) numerically and obtain the scaled activity from Eq (6) (blue curve in Fig 4(a)). Subsequently, the *cellular responsivenesses* $\alpha_{R}^{gl}$ and $\alpha_{R}^{inj}$ are computed separately by integrating over different parts of the scaled activity and scaling them with *T*_*gl*_ and *T*_*inj*_ for $\alpha_{R}^{gl}$ and $\alpha_{R}^{inj}$ in Eq (8), respectively. The resulting *cellular responsiveness* is computed as $\alpha_{R}=\alpha_{R}^{gl}\left(L\right)+\alpha_{R}^{inj}\left(L\right)$.
- model 2: Eq (1) is solved separately for both, *L*_*gl*_ and *L*_*inj*_. Then, the *cellular responsiveness* is computed as $\alpha_{R}=\alpha_{R}^{gl}\left(L_{gl}\right)+\alpha_{R}^{inj}\left(L_{inj}\right)$, see Fig 4b.

Table 2 indicates that both models produce nearly the same *cellular responsiveness* *α*_*R*_ results for different doses and illnesses. Therefore, in the following computation, the computationally less expensive model 2 is utilised.

**Table 2:** Computed *cellular responsiveness* for different PTH doses using model 1 and model 2.

daily drug dose *D*	healthy	idiopathic osteoporosis	hyperparathyroidism
0*μ*g	0.939/0.939 (0)	0.497/0.497 (0)	2.105/2.105 (0)
10*μ*g	1.358/1.398 (0.459)	0.957/0.957 (0.459)	2.376/2.443 (0.338)
20*μ*g	1.778/1.818 (0.879)	1.375/1.376 (0.879)	2.694/2.835 (0.730)
30*μ*g	2.143/2.183 (1.244)	1.759/1.741 (1.244)	3.042/3.179 (1.074)

Computed *cellular responsiveness* for different PTH doses administered via sc daily injections (model 1/model 2) superposed to baseline glandular secretion patterns. The results are given in form model 1 /model 2 ($\alpha_{R}^{inj}$). For example, considering a healthy person and a PTH dose of 20*μ*g, *α*_*R*_ computed from model 2 is obtained as a sum 0.939 + 0.879 = 1.818.

## 3 Optimal pulsatile regimes to reach maximal or targeted *cellular responsiveness*

We note that in the original work [16], a non-constrained optimisation for a square-wave signal with constant *γ*_0_ and *γ*_1_ was utilised. This is based on an analytical formula for an optimal *τ*_1_ and *T*. However, as the numerical solution is available, different dosing patterns, i.e. combination of glandular and drug contribution (see Section 3.2) and different optimisation questions in addition to the maximisation of the *cellular responsiveness* can be answered. After transformation of the constrained optimisation problems to equivalent unconstrained ones, all optimisation problems are solved with the function *fminsearch* in MATLAB. In the following, we describe the formulation of different optimisation problems.

### 3.1 Baseline PTH secretion

As described earlier, the tonic secretion makes up approximately 70% and the pulsatile secretion the remaining 30%. Based on the data in [17, 18], the integrated concentration remain in the same range and hence we assume that the AUC in the (*L*, *t*)-plot can be rewritten as *A* = *A*_*tonic*_ + *A*_*puls*_. To fix the mean plasma concentration at the reference value obtained from experimental data, we enforce that *A* = *A*^*ref*^. In order to predict the optimal pulsatile pattern to reach the maximum *cellular responsiveness* $\alpha_{R}^{max}$, we consider the following constrained optimisation problem

max(τ1,T,γ0,γ1)αR(Lgl(τ1,T,γ0,γ1,t))withtheconstraints{A(s,Lgl)=Aref(s,Lgl)Apuls=rA (15)

where *r* is the ratio of pulsatile integrated concentration to total integrated concentration (see S1 Table), $A\left(s\right)=\int_{0}^{s}L_{gl}\left(\tau_{1},T,\gamma_{0},\gamma_{1},t\right)\text{d}t$ is the AUC in a (*L*_*gl*_, *t*)-plot during an arbitrary time interval [0, *s*] and the superscript *ref* refers to the quantities based on the experimental data for a healthy person [17, 18]. With the assumed square-wave stimulus in Eq (10) follows $A\left(s\right)=\gamma_{0}s+\frac{\left(\gamma_{1}-\gamma_{0}\right)\tau_{1}s}{T}$. The constraints in Eq (15) yield then

$$\gamma_{0}=\gamma_{0}^{ref}$$ (16)

$$T=s\tau_{1}\left(\gamma_{1}-\gamma_{0}\right)/rA^{ref}$$ (17)

Therefore, the constrained optimisation problem in Eq (15) can be reduced to a non-constrained optimisation problem

max(γ1,τ1)αR(Lgl(τ1,T,γ0,γ1,t)) (18)

with *γ*_0_ and *T* according to Eqs (16) and (17).

Another important question that can be addressed using the proposed optimisation method is “Can a healthy *cellular responsiveness* $\alpha_{R}^{ref}$ be reached by manipulating the glandular secretion pattern?”. Note that the case of external PTH injection is considered below. Here, we explore the possibility of modulating a pathological glandular secretion pattern such that one would obtain *α*_*R*_ close to the healthy case. To answer the question if it is possible to reach the *cellular responsiveness* of the healthy person solely by changing the pulsatile pattern and keeping the area *A* constant, the following minimisation problem is to be solved

min(γ1,τ1)(αR(Lgl(τ1,T,γ0,γ1,t)-αRref))2 (19)

### 3.2 PTH injection

The previous concept can be applied to the investigation of the optimal dosing regimes for sc PTH injection and furthermore extended to other drugs. Here, we remark that *τ*_1_ and *γ*_1_ are not independent as they are given by the injected dose of the ligand and the experimentally measured pharmacokinetics. This is approximated by a function *f*_*PK*_(*γ*_0_, *D*) determining the corresponding values, i.e. (*γ*_1_, *τ*_1_) = *f*_*PK*_(*γ*_0_, *D*). For different PTH daily doses (*D* ∈ {10*μg*, 20*μg*, 30*μg*}), *cellular responsiveness* is computed exemplarily for healthy person and patients with idiopathic osteoporosis and hyperparathyroidism.

#### 3.2.1 Maximising the *cellular responsiveness*

Let *A*_*inj*_ be the AUC in the (*L*, *t*)-plot resulting from the sc injection of PTH (see Fig 4) and $A_{inj}^{ref}$ the reference area corresponding the clinically used doses of 20*μ*g once daily. The optimisation problem becomes

max(D,T)αRinj(D,T,γ0)withtheconstraintAinj(D,T,γ0)=Ainjref. (20)

which again is equivalent to a non-constrained optimisation

maxDαRinj(D,T,γ0) (21)

with $T=\tau_{1}\left(\gamma_{1}-\gamma_{0}\right)/A_{inj}^{ref}$ and (*γ*_1_, *τ*_1_) = *f*_*PK*_(*γ*_0_, *D*) are given by the PK model [37].

#### 3.2.2 Targeting healthy *cellular responsiveness* using external PTH injections

Suppose that $\alpha_{R}^{ill}$ denotes an altered *cellular responsiveness* compared to a healthy reference value $\alpha_{R}^{ref}$, e.g. for patients with osteoporosis. Note that this could be higher or lower than $\alpha_{R}^{ref}$. We are aiming to find a dose *D* such that the *cellular responsiveness* of the healthy person is reached, i.e. we solve the following optimisation problem

minD(αRinj(D,T,γ0)-(αRref-αRill))2. (22)

This gives the optimal dose to normalise the PTH *cellular responsiveness* by keeping the dosing period unchanged (*T* = 24 h).

## 4 Results of numerical simulations

### 4.1 Basal secretion

**Fig 5:**
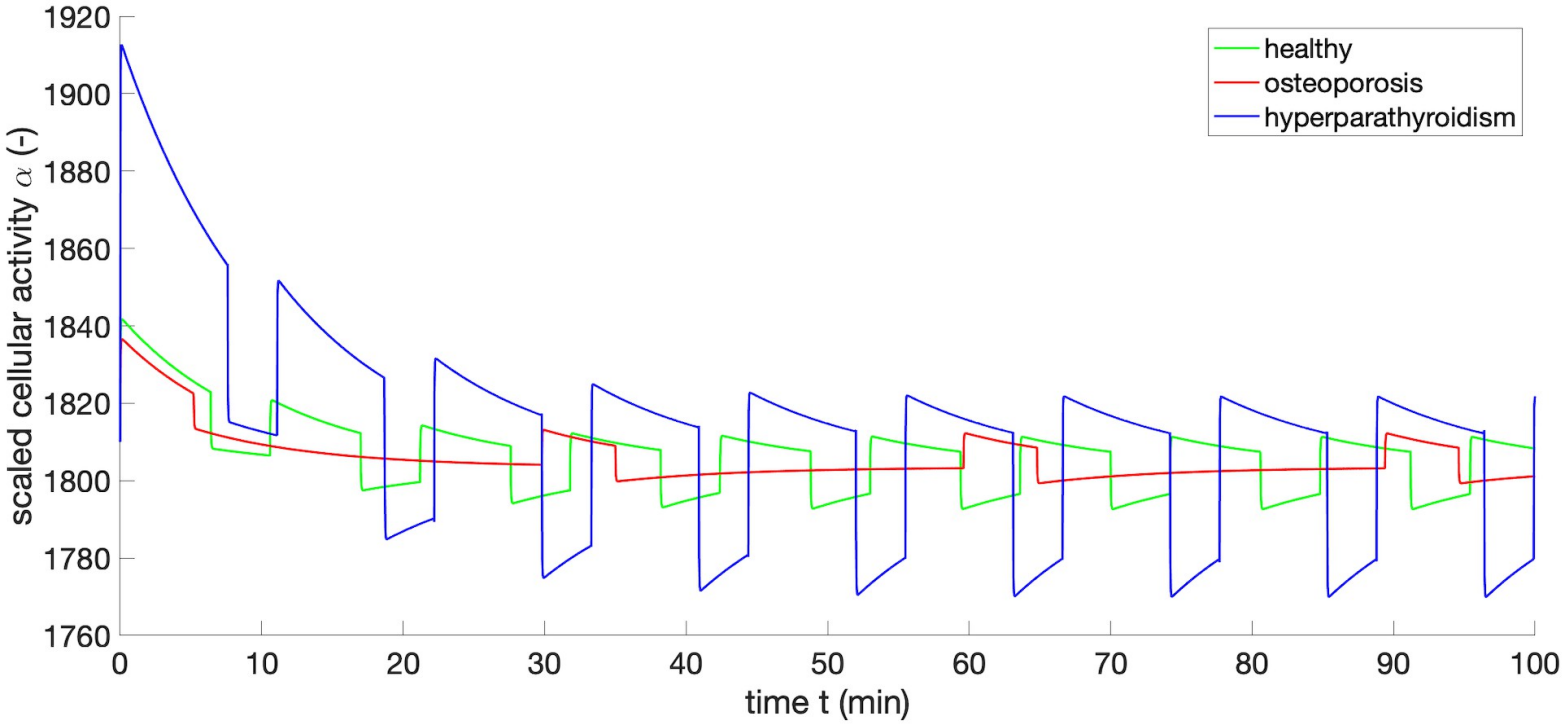
Scaled activity *α* for healthy person, patient with idiopathic osteoporosis and hyperparathyroidism. Simulation parameters are given in Table 1 and S2 Table.

Table 3, first row, shows computed *α*_*R*_ using the mean experimental data [8, 17, 18] for healthy people and patients with different types of osteoporosis, hyperparathyroidism as well as under hyper- and hypocalcemia conditions. We note that if the tonic secretion is reduced in a healthy person, then the *cellular responsiveness* is slightly higher than the reference value. The corresponding scaled activity *α* computed according to Eq (6) is displayed in Fig 5, exemplarily for three cases with reference, reduced and increased *α* values—healthy, idiopathic osteoporosis and hyperparathyroidism, respectively.

**Table 3:** *Cellular responsiveness* for gland secretion.

parameter	healthy [17], r	OP	PMO [38, 39]	GIO	HP	hypocal 1	hypocal 2	hypercal
*α*_*R*_	0.939/0.957	0.497	0.939/0.856	1.607	2.105	4.645	1.814	0.174
$\alpha_{R}^{max}$	3.345/3.384	0.649	3.345/2.757	5.689	9.983	27.653	6.798	0.466
$\tau_{1}^{max}$ (min)	12.92/12.89	11.77	12.92/12.64	13.49	14.78	15.67	14.24	11.33
*T*^*max*^ (min)	54.94/54.31	65.94	55.94/55.57	51.23	55.04	50.01	55.12	65.78
$\gamma_{1}^{max}$ (pm/L)	10.41/9.68	4.98	10.41/8.52	12.67	38.73	78.18	25.63	1.99
$\tau_{1}^{*}$ (min)	6.40/6.54	-	6.40/6.46	6.33	7.80	-	6.42	-
T* (min)	10.60/10.69	-	10.60/11.50	8.94	9.80	-	8.84	-
$\gamma_{1}^{*}$ (pm/L)	6.08/5.38	-	6.08/5.02	5.71	22.21	-	15.45	-

*Cellular responsiveness* *α*_*R*_ for gland secretion computed according to Eq (8), *α*_*R*_ and the corresponding *τ*_1_, *T*, *γ*_1_ are denoted with the superscripts *max* and * as solutions of the optimisation problems in Eqs (18) and (19), respectively. Coding: healthy = healthy person [17], r = healthy with reduced (by 20%) tonic secretion, OP = idiopathic osteoporosis [17], PMO = postmenopausal osteoporosis based on [38, 39], GIO = glucorticoid-induced osteoporosis [40], HP = hyperparathyroidism [18], hypocal 1 = hypocalcemia initial state [32], hypocal 2 = hypocalcemia steady state [32], hypercal = hypercalcemia steady state [32]. - optimisation problem (19) has no solution within the tolerance. Simulation parameters are given in Table 1 and S2 Table.

In Table 3, solutions of the optimisation problems given in Eqs (18) and (19) are summarised. Superscript *max* denotes the values yielding the maximal *cellular responsiveness* by conserving the area *A*. Superscript * refers to the optimal values needed to reach the *cellular responsiveness*s of a healthy person. We note that this is possible only if $\alpha_{R}^{max}\geq \alpha_{R}^{ref}$.

**Fig 6:**
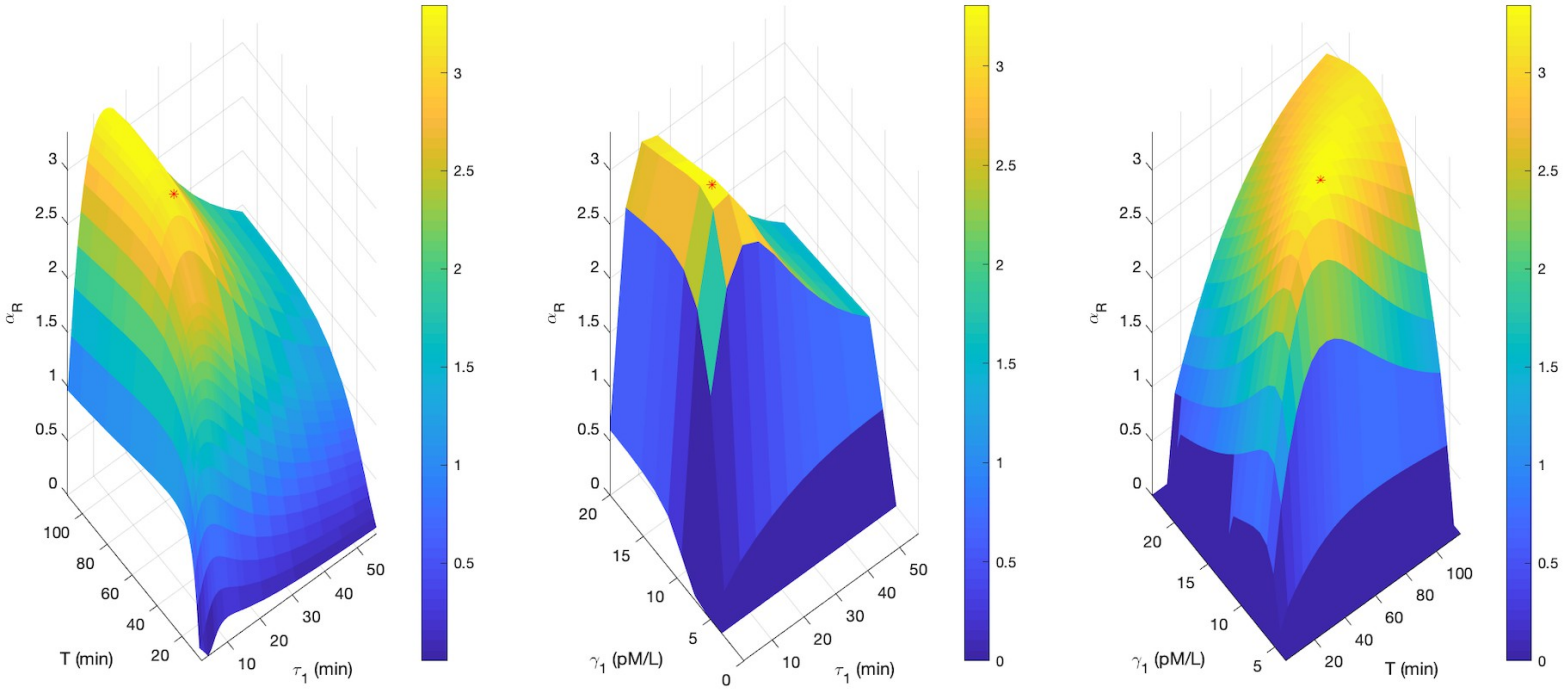
*Cellular responsiveness* for a healthy person as function of *T* and *τ*_1_; *γ*_1_ and *τ*_1_; *γ*_1_ and *T*. The third quantity of the triplet (*γ*_1_, *τ*_1_, *T*) results from the optimisation constraint given in Eq (15). Simulation parameters are given in Table 1 and S2 Table.

Fig 6 shows surface plots where *α*_*R*_ for a healthy person is a function of two variables of the triplet (*τ*_1_, *T*, *γ*_1_). The third component is computed from Eq (17). The maximal *cellular responsiveness* is in each plot labelled with a red star. Its value $\alpha_{R}^{max}=3.35$ is for all three cases identical because the same constrained optimisation problem given in Eq (15) is solved.

### 4.2 PTH external injection

**Fig 7:**
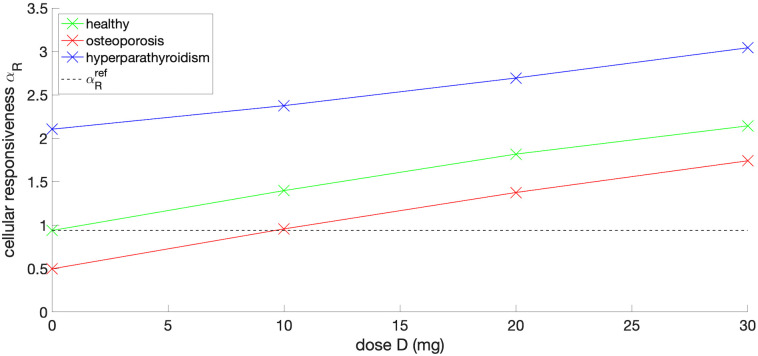
*Cellular responsiveness* computed with model 2 for healthy person, patients with idiopathic osteoporosis and hyperparathyroidism after different doses of PTH sc injection are applied once daily.

In the following, simulation results for various PTH dosing patterns are presented with the aim of identifying optimal dosing regimes leading to maximum cellular responsiveness. Further, an optimal PTH dose of 9.61*μ*g daily has been computed in order to reach the target *cellular responsiveness* of a patient with osteoporosis, see Table 4. The intersection of the red and black dashed lines in Fig 7 visually confirms that the optimal dose is close to 10*μ*g. The latter figure also indicated that the *cellular responsiveness* increases nearly linearly as the daily dose increases.

**Table 4:** Maximum *cellular responsiveness* for PTH doses administered via sc daily injections.

parameter	10*μ*g	20*μ*g	30*μ*g	optimised dose according to Eq (22)	
$\alpha_{R}^{max}$	1.455	2.183	2.817	$\alpha_{R}^{ref}/\alpha_{R}^{ill}$	0.939/0.497
*D*^*max*^ (*μ*g)	1.606	2.866	4.049	D*	9.61
$\tau_{1}^{max}$ (min)	43.30	58.76	67.67	$\tau_{1}^{*}$	87.90
*T*^*max*^ (min)	79.18	98.56	107.60	*T*	1440
$\gamma_{1}^{max}$ (pM/L)	6.63	8.51	10.32	$\gamma_{1}^{*}$	17.16

Maximum *cellular responsiveness* for PTH doses administered via sc daily injections given to a patient with osteoporosis. Model 2 is used for optimisation. Superscript *max* denotes values leading to maximum *cellular responsiveness* $\alpha_{R}^{max}$ by keeping the daily drug dose, i.e. the area *A*_*inj*_ unchanged. Superscript * denotes the optimal values to reach the reference *cellular responsiveness* $\alpha_{R}^{ref}$.

## 5 Discussion

In this paper, we utilised a two-state receptor model to investigate bone cellular responses due to PTH glandular secretion patterns together with external administration of PTH. We considered five different types of glandular secretion patterns corresponding to healthy subjects and patients with osteoporosis, hyperparathyroidism, hypercalcemia and hypocalcemia. Our model a priori prescribes a particular glandular PTH secretion pattern reported in the literature and then calculates the corresponding cellular activity. We note that the PTH glandular secretion plays a pivotal role in calcium and phosphorus homeostasis and, consequently, factors that affect the different organs involved in controlling mineral balance can also influence the glandular secretion patterns. Major factors that can affect PTH glandular secretion are dietary conditions (i.e., calcium and phosphorus intake), lifestyle (i.e., physical activity), and genetics (i.e., vitamin D system). However, it is beyond the scope of the current study to investigate the effects of these factors on glandular PTH secretion patterns.

Using an efficient numerical approach to solve the ODEs allowed us to formulate a variety of optimisation problems which aimed at restoring the perturbed bone cellular response due to different disease back to the healthy baseline state. We note that the computation of the *cellular responsiveness* based on a two-state receptor model is for the first time computed numerically. The numerical approach is much more flexible in the sense that there are no special case restrictions on the parameters in the model and one can easily consider different scenarios by formulating constrained optimisation problems with various objective functions and constraints. The MATLAB codes are provided on request.

Below, we discuss first how the different bone cell response values can be interpreted in a bone remodelling context. Then, we discuss the manipulation of glandular secretion patterns and the superposition of an external PTH injections. Finally, we will also discuss the limitations of the current study.

### 5.1 Comparison of healthy versus disease states

While the bone cellular response parameter (*α*_*R*_) of the current two-state receptor model is not linked to any (downstream) catabolic or anabolic bone cell responses, it is instructive to view this parameter’s action on the bone remodelling process. In the bone remodelling models of Lemaire et al. and Pivonka et al. [41–43], a single monovalent binding reaction function was used to simulate the catabolic action of PTH on bone remodelling. This was achieved by introducing a PTH activator function which was a sigmoidal function of the PTH serum concentration mapped on the interval 0 and 1. Using this function, the catabolic ligand (RANKL) expressed on osteoblastic cells was monotonously increased for pathological cases of increased PTH serum concentration. We note that serum PTH concentration was assumed constant in these models and based on the rather simplistic receptor ligand binding model, it was emphasised that these models are not able to capture pulsatile effects of PTH such as the anabolic response due to external intermittent PTH injections. Hence, a reduction in serum PTH concentration gave rise to reduced bone remodelling with modest to no anabolic bone gains.

In the following, we interpret the decrease or increase of *α*_*R*_ with respect to catabolic actions of bone cells on the remodelling process. Hence, an increased value of the bone cellular response parameter (compared to the healthy baseline value) indicates a catabolic response, while a decreased value of *α*_*R*_ indicates a moderate anabolic bone response. With this in mind, the simulation results for hyperparathyroidism and hypocalcemia indicate that for the given pattern of glandular PTH secretion, a catabolic bone response is obtained. Hence, for these pathological conditions, net increase in bone resorption will occur. Primary hyperparathyroidism is due to a benign overgrowth of parathyroid tissue either as single gland (80% of cases) or as a multiple gland disorder. For symptomatic cases, it is associated with bone loss. The latter can be treated by using antiresorptive drugs, but more often hyperparathyroidism can be cured by removing the parathyroid gland(s) [6].

For the hypocalcemia simulations representing the hypocalcemic clamp tests reported in [32], we found that the PTH glandular secretion pattern in the transitional period of hypocalcemia (i.e., hypocalcemia 1) gave rise to a very high *α*_*R*_ value indicating high bone resorption, while the *α*_*R*_ value for the steady state period was significantly lower indicating lower bone resorption. Based on the tight regulation of calcium homeostasis, these results are meaningful, i.e. the immediate reduction of calcium due to the clamp test is rapidly compensated for by high bone resorption followed by a more modest bone resorption period.

For the hypercalcemia simulations representing the hypercalcemic clamp test reported in [32], we found that the PTH glandular secretion pattern led to a lower value of *α*_*R*_ and consequently reducing bone remodelling. During bone remodelling calcium is released from the bone matrix and, consequently, reducing bone remodelling avoids release of calcium into the plasma [44].

For the case of the osteoporosis simulations, lower, approximately similar and higher *α*_*R*_ values compared to healthy baseline were obtained for the idiopathic osteoporosis (OP), postmenopausal osteoporosis (PMO) and glucocorticoid-induced osteoporosis (GIO) case, respectively. Below we interpret these results in more detail. The results for the idiopathic OP seem to be not physiologically meaningful given that osteoporosis is associated with bone loss (i.e., catabolic bone response) [45]. However, there are quite a number of things to consider for interpretation of this result. The OP data [17] are based on only three male patients with idiopathic OP (mean age, 37 yr; range, 31–42 yr). This is an extremely low sample number. Also, research into establishing a link between PTH patterns and other types of osteoporosis indicate that PTH modulation may be only a secondary effect, but that other catabolic bone regulators (such as RANKL) could directly be affected by the disease. Below, we discuss some of these findings.

For cases of PMO, our simulation results indicate that *α*_*R*_ is equal or slightly reduced compared to the reference value. These results are based on qualitative data provided in [8] for describing the PTH glandular secretion pattern in PMO. However, the data on this pattern are not conclusive as discussed next. Samuels et al. [38] compared PTH glandular secretion patterns from healthy young subjects with postmenopausal women of various oestrogen levels and bone mineral density (BMD). They found there were no differences in the amplitude or frequency of pulsatile PTH secretory parameters between the pathological cases and the healthy group. They concluded that different types of PMO do not alter PTH secretory patterns and temporal organisation. Based on these findings, the authors suggested that abnormalities in orderly pulsatile PTH secretion are unlikely to play a major role in driving PMO related osteoporosis. We note that oestrogen deficiency, i.e. a hallmark of PMO, has been directly linked with increased RANKL production.

For the case of GIO, which is the largest cause of secondary osteoporosis, some studies have not found an association between GIO and increased PTH concentration. On the other hand, other studies have shown that patients with GIO did exhibit elevated PTH serum concentrations [46]. However, as pointed out in [8], the PTH serum levels have little meaning in identifying a role of PTH in driving GIO, but it is the glandular pulsatile secretory pattern of PTH that might provide a link. However, only a few studies have investigated this pattern in a very small population of men. Bonadonna et al. [40] evaluated spontaneous PTH pulsatile secretion in patients chronically treated with pharmacological amounts of glucocorticoids. Their findings indicate that in the glucocorticoid-treated group, the PTH tonic secretory rate was reduced, while there was an increase in the fractional pulsatile PTH secretion in glucocorticoid-treated vs normal subjects. Mean overall PTH concentration, as well as mean integrated area, was similar among normal and glucocorticoid-treated subjects. Therefore, it can be concluded that chronic glucocorticoid treatment induces a redistribution of spontaneous PTH secretory dynamics by reducing the amount released in tonic fashion and increasing the amount released as pulses. In this study, GIO is simulated by scaling the parameters *τ*_1_, *T*, *γ*_0_ and *γ*_1_ according to the data provided in [40], see S2 Table. In particular, the tonic secretion is nearly halved while the pulsatile pattern remains similar as the reference values. Our simulation produces *α*_*R*_ value significantly larger than in the healthy case. This indicates bone resorption which is in agreement with the obtained findings.

### 5.2 Optimisation of glandular dosing patterns to restore healthy cell response

Our simulation results for some of the PTH glandular disease states indicate that it may be possible to pharmacologically manipulate the PTH secretion pattern such as to restore similar values for *cellular responsiveness* as for healthy subjects. This would indicate a similar bone remodelling behaviour. We note that this could only be achieved for pathological cases that led to $\alpha_{R}^{max}$/$\alpha_{R}^{min}$ values higher/lower than the healthy baseline value, i.e. PMO, hyperparathyroidism, hypocalemia clamp test, and GIO. These cases, except PMO, could be restored towards the healthy $\alpha_{R}^{ref}$ by manipulating the pulsatile profile to a more continuous pattern which desensitises the active receptors and ligand complexes and leads to lower values of *α*_*R*_. For PMO, solely the manipulation of the pulsatile pattern leads to an elevation of *α*_*R*_ up to the reference value. On the other hand, PTH glandular diseases that led to maximum bone *cellular responsiveness* below the healthy baseline value such as the idiopathic osteoporosis case reported by Harms et al. [17], can’t be restored to baseline via glandular manipulation. For such cases, external PTH injections are a viable solution which is further discussed below. Moreover, in the case of the initial hypercalcemia, the minimum *cellular responsiveness* $\alpha_{R}^{min}$ is higher than $\alpha_{R}^{ref}$ and therefore a lowering to the targeted level is not possible, too.

### 5.3 Effects of external PTH injections

To simulate the effect of external sc once daily PTH administration, as it is currently clinically available, one had to superpose the external PTH injection with the glandular PTH secretion. We explored two modelling approaches: In model 1, we assume that the resulting PTH ligand concentration in Eq (1) becomes *L* = *L*_*gl*_ + *L*_*inj*_. Subsequently, Eq (1) is solved numerically. In model 2, Eq (1) is solved separately for both, *L*_*gl*_ and *L*_*inj*_. Then, the *α*_*R*_ is given as a sum of the *cellular responsiveness* of the gland and a sc injection. Comparison of the simulation results for these two models indicated that the final values for *α*_*R*_ are very similar. Given that the computational efficiency of model 2 was significantly higher than model 1, all optimisations were performed for model 2. A general observation is, that superposing external PTH injections onto a baseline glandular secretion gave rise to increased values of *α*_*R*_. The increase of the *cellular responsiveness* scaled nearly linearly with respect to the external PTH dose. Hence, for the case of osteoporosis, optimum external PTH dose of *D* = 10*μ*g daily injections, that would give a *cellular responsiveness* close to the healthy state.

### 5.4 Limitations of the current study

The current study has a number of limitations. The receptor ligand PTH-PTH1R binding constants are taken from the open literature. We note that we deliberately did not use the constants that were proposed in [15] for several reasons. Firstly, the constants are not experimentally determined and do not fulfil the condition of the detailed balance as proposed [16]. Secondly, using the constants suggested in [15] gives several orders of magnitude difference between the active complexes and inactive complexes, which seems physiologically not realistic.

Another limitation of our study is that we discussed mainly the action of PTH on RANKL expression on cells of the osteoblastic lineage with no discussions of potential feedback mechanisms in the organs responsible for mineral metabolism. One such factor is fibroblast growth factor 23 (FGF-23) which is most highly expressed in bone, predominantly in osteocytes [47, 48]. In healthy subjects FGF-23 is expressed at low levels in osteocytes, but is significantly increased in osteocytes in patients with hypophosphatemic rickets [49] and in patients with chronic kidney disease [50]. It has been suggested that circulating FGF-23 directly acts on parathyroid glands to modify PTH dosing pattern [51].

We acknowledge that the current study exclusively focused on the PTH-PTH1R system that is most relevant for regulation of bone metabolism. We note that PTH also binds to its second receptor PTH2R together with its Ligand Tuberoinfundibular Peptide of 39 Residues (TIP39) to particularly regulate skin function via differentiation of keratocytes [52].

## 6 Conclusions

We developed a novel two-state PTH-PTH1R receptor binding model to analyse the effects of PTH glandular secretion patterns on bone *cellular responsiveness*. In particular, we explored differences between healthy and pathological glandular secretion patterns and their effect on bone cellular responsiveness. We explored the potential of (pharmacological) manipulation of pathological glandular secretion patterns back to its normal baseline bone cellular responsiveness. Also, we investigated the effect of (external) once daily PTH injections on bone cellular responsiveness. For this purpose, we formulated a variety of constrained optimisation problems with and without keeping the area under the curve constant. Based on our numerical simulations, we found the following:

- Bone *cellular responsiveness* in healthy subjects is sensitive to the tonic baseline stimulus and is significantly below the maximum responsiveness;
- Bone *cellular responsiveness* greatly varies between healthy and pathological glandular secretions patterns;
- Catabolic bone diseases, as defined by $\alpha_{R}^{ill}>\alpha_{R}^{ref}$, (with exception of very high hypocalcemia) could be restored to normal by manipulating the pulsatile component of glandular secretion of the pathological gland;
- Bone diseases characterised by $\text{max}\left(\alpha_{R}^{ill}\right)<\alpha_{R}^{ref}$ with a maximum *cellular responsiveness* value below healthy baseline can’t be restored to baseline.
- External once daily PTH injections can restore the above cases back to normal.
- Superposition of glandular PTH secretion and external PTH injections can be effectively achieved by additive superposition;
- Based on the numerical formulation of the two-state receptor model, constrained optimisation problems can be efficiently formulated and solved;

In conclusion, the current two-state receptor model seems to capture major aspect of PTH glandular secretion and associated skeletal pathologies. In the future, this type of model could be used for analysing pharmacological drug effects on glandular secretion.

## Supporting information

S1 Table Experimental data. Experimental data for PTH, based on the cited literature. The values are given as mean ± sd or median (minimum—maximum).
(PDF)

S2 Table Simulation parameters. Simulation parameters for cases: healthy person [17]/healthy with reduced (by 20%) tonic secretion, idiopathic osteoporosis (OP), postmenopausal osteoporosis (PMO), glucocorticoid induced osteoporosis (GIO), initial hypocalcemia (hypocal 1), steady state hypocalcemia (hypocal 2) and hypercalcemia (hypercal) and glucocorticoid induced osteoporosis (GIO). The parameters for healthy person, OP and HP are based on the experimental provided in [17] which are shown in S1 Table. The remaining parameters are obtained via scaling the experimental data for healthy people [17] with relative changes presented in [38–40, 53] for PMO, GIO and different calcium levels, respectively.
(PDF)
