# Supplementary material for: Effects of PTH glandular and external dosing patterns on bone cell activity using a two-state receptor model—Implications for bone disease progression and treatment
Source: PLoS One. 2023 Mar 30;18(3):e0283544. doi: 10.1371/journal.pone.0283544 (PMC10062658; doi:10.1371/journal.pone.0283544)
Supplement: S1 Table — Experimental data for PTH, based on the cited literature. The values are given as mean ± sd or median (minimum—maximum). (PDF) [file pone.0283544.s001.pdf]

parameter	[17, 18] healthy/osteoporosis/hyperparathyroidism	[33]	[35] men/women
τ_1 (min)	$6.4 \pm 1.3/5.2 \pm 2.4/7.6 \pm 1.6$	6.5	-
T (min)	$10.6 \pm 1.3/29.8 \pm 28.7/11.1 \pm 2.5$	8.7 ± 1.1	$21 \pm 2.0/23 \pm 2.0$
mean plasma concentration $\bar{L}_{gl}$ (pM/L)	$5.0 \pm 1.0/3.6 \pm 2.0/20.5 \pm 13.6$	$3.2\ (2.7 - 5.0)$	$2.4 \pm 0.4/2.8 \pm 0.4$
pulsatile peak amplitude $\gamma_1 - \gamma_0$ (pM/L)	$2.8 \pm 1.7/1.7 \pm 1.3/9.8 \pm 7.4$	-	-
A_{tonic} per hour (pM/Lh)	199.3/198.2/825.0	-	-
A_{puls} per hour (pM/Lh)	100.7/17.8/405.0	-	-
total tonic secretion per hour (pM/Lh)	-	38.9 ± 12.6	$32.0 \pm /35.6 \pm$
total pulsatile secretion per hour (pM/Lh)	-	17.5 ± 6.1	$9.9 \pm /13.7 \pm$
ratio pulsatile to total concentration/secretion $r 100\%$ (%)	33.5/8.2/32.9	38.9	23.6/27.8
