# Supplementary material for: Effects of PTH glandular and external dosing patterns on bone cell activity using a two-state receptor model—Implications for bone disease progression and treatment
Source: PLoS One. 2023 Mar 30;18(3):e0283544. doi: 10.1371/journal.pone.0283544 (PMC10062658; doi:10.1371/journal.pone.0283544)
Supplement: S2 Table — Simulation parameters for cases: healthy person [17]/healthy with reduced (by 20%) tonic secretion, idiopathic osteoporosis (OP), postmenopausal osteoporosis (PMO), glucocorticoid induced osteoporosis (GIO), initial hypocalcemia (hypocal 1), steady state hypocalcemia (hypocal 2) and hypercalcemia (hypercal) and glucocorticoid induced osteoporosis (GIO). The parameters for healthy person, OP and HP are based on the experimental provided in [17] which are shown in S1 Table. The remaining parameters are obtained via scaling the experimental data for healthy people [17] with relative changes presented in [38–40, 53] for PMO, GIO and different calcium levels, respectively. (PDF) [file pone.0283544.s002.pdf]

parameter	healthy [17]/ r	OP	PMO [39]/[40]	GIO	HP	hypocal 1	hypocal 2	hypercal
τ_1 (min)	6.4/6.4	5.2	6.4/6.4	6.1	7.6	3.2	6.1	9.4
T (min)	10.6/10.6	29.8	10.6/10.6	10.1	11.1	5.4	10.2	15.6
γ_0(pM/L)	3.32/2.65	3.32	2.65/3.32	1.59	13.81	8.53	9.82	0.83
γ_1 (pM/L)	6.08/6.08	5.01	4.86/6.08	6.42	23.58	36.16	6.76	0.69
